# Exploring Living Arrangements as a Predictor of Canadians’ Illicit Drug Use: Quantitative Findings From the Canadian Community Health Survey

**DOI:** 10.3389/ijph.2023.1605619

**Published:** 2023-05-10

**Authors:** Xiangnan Chai, Liu Liu, Guangli Huang, Yongzhen Tan

**Affiliations:** ^1^ Department of Sociology, School of Social and Behavioral Sciences, Nanjing University, Nanjing, China; ^2^ Department of Social Work and Social Policy, School of Social and Behavioral Sciences, Nanjing University, Nanjing, China

**Keywords:** Canadian adults, living arrangements, illicit drug use, health behaviour, gender difference, health lifestyle theory

## Abstract

**Objectives:** About four percent of Canadians used illegal drugs in 2019, but it remains unknown whether their living arrangements are a relevant factor.

**Methods:** We use the public version of the 2015–2016 Canadian Community Health Survey Annual Component. The binary logit model and complementary log-log model are applied to investigate to what extent living arrangements predict Canadians’ recent illicit drug use.

**Results:** Living alone is significantly associated with Canadians’ illicit drug use. For young and older Canadians, those living with spouses/partners, children, or both are less likely to use illicit drugs than their solo-living counterparts. Middle-aged Canadians who lived with spouses/partners only or with children have significantly lower likelihoods of using illicit drugs compared to those living alone. Additionally, differences between men and women have been found. Spouses/partners and children play more positive roles for young and middle-aged women than for men.

**Conclusion:** Our findings suggest that living with core families is a type of collectivity that may have positive effects on Canadians’ health behaviours compared to those living alone, who, therefore, need more attention from health officials.

## Introduction

Illicit drug use has long been a social issue in Canada. Although personal use of cannabis has been legalized in the country since 17 October 2018, under the *Cannabis Act* (1), many other drugs remain illegal but are still prevalent. A recent report demonstrates that around four percent of Canada’s total population once used illicit drugs in 2019, including “cocaine, ecstasy, methamphetamine, hallucinogens, inhalants, heroin and salvia” (2). The age difference is further found in illicit drug use among Canadians—those between 20 and 24 years of age have the highest proportions of using illegal drugs compared to younger or older individuals (3).

Researchers debate whether illicit drug use leads to detrimental health behaviours and mental health problems (4). Despite the debate, prior empirical studies in Canada (5), the U.S. (6), the U.K. (7), EU countries and Norway (8), and China (9) demonstrate that using illicit drugs is a public health burden. This is due mainly to the significant association with detrimental health outcomes, such as infectious disease transmission and overdose death. Some studies have further identified that indirect mechanisms, for example, discrimination and social exclusion due to drug addiction, may also contribute to worse mental health among users (10).

Living arrangements have been examined as a factor related to people’s illicit drug use behaviour, but details remain underexplored. Prior studies in some other countries have found that young adults who live by themselves are more likely to use illicit drugs (11, 12), while living with one’s parents is associated with a lower likelihood of doing so (13). However, there are two unanswered questions. First, compared to living alone, is living with different types of family members associated with a higher or lower probability of using illicit drugs? Second, do these associations differ between men and women? Further exploration is urgently necessary because people’s living arrangements have shifted considerably in Canada and many other high-income countries.

### Living Arrangements and its Complex Association With Health Behaviours

Living arrangements are associated with people’s health behaviours. Living with families may influence people’s health behaviours through mutual interactions (14), but the direction could be complex. Contradictory findings have been found concerning the association between living arrangements and health behaviours, showing the complexity of this subject. Compared to those living with families, people who live alone may have higher likelihoods of eating unhealthily (15), smoking cigarettes, and consuming alcohol (16). They may also have worse physical and mental health (17), and lower levels of life satisfaction (18). However, some studies found no significant differences in some health dimensions between living alone versus living with families. For example, older adults living alone do not differ regarding time spent engaging in physical activities from their counterparts co-residing with families (19).

Canadians’ living arrangements have shifted remarkably over the past four to five decades, and are still in a consistent transition. Specifically, single-person households now make up the highest proportion of all household types (20). This transition is mainly due to compositional changes at the population level in Canada’s sex ratios, the rising levels of Canadians’ educational attainments, the increasing proportion of women’s labour force participation, people’s changing marriage and fertility behaviours, and its ageing population structure (17, 21). Under this context, however, to date, little scholarly attention has been paid to identifying whether living arrangements are associated with Canadians’ illicit drug use.

### Health Lifestyle Theory: Highlighting the Effect of Collectivities

We apply the health lifestyle theory, which is widely used in sociology, to explore how essential others, usually family members, may have influenced people’s illicit drug use behaviours. As the main theoretical contributor, Cockerham (p.6) (22) defines health lifestyles as “collective patterns of health-related behaviour based on choices from options available to people according to their life chances.” Health lifestyle theory aims to explain how structural factors contribute to the production and reproduction of people’s health lifestyles. Specifically, Cockerham (23) points out that interactions and interplay between structural factors and people’s agency they learned and practised during past life experiences produce dispositions to act, which affect their daily actions and further shape health lifestyles. Structural factors proposed by Cockerham et al. (24) are factors at the levels of social institutions or conditions that exert influences on individuals’ health behaviours and health lifestyles, including social class, demographic background (e.g., age, gender), living conditions, and collectivities.

The current research does not address an individual’s agency but aims to investigate social structure as a potential influential facet to the production of health behaviours and health lifestyles. Our emphasis is to test living arrangements as a type of collectivity. From a theoretical lens, collectivities are “collections of actors linked together through particular social relationships, such as kinship, work, religion, and politics” (22). Living arrangements refer to whom an individual is living with. Living arrangements imply daily interactions and mutual influences among family members regarding health-promoting or health-harming behaviours and lifestyles. Norman and Ford (25) indicate that close ties with families or other essential others significantly increase people’s social involvement and decrease the possibility of deviant or unhealthy behaviours. For example, Tucker and Anders (26) contend that married couples are likely to promote changes in health behaviours for their partners, such as healthy eating and physical exercise. On the contrary, a recent Japanese study indicates that older men living alone are more likely to eat alone, which is associated with unhealthy eating and obesity (27).

Differences between men and women are also widely reported in terms of mutual influences on health behaviours within the household. Women are more likely to encourage healthy behaviours (28) and discourage unhealthy behaviours (28, 29), leading to men benefitting more from their female partners rather than the opposite. For example, Margolis (30) investigated the changes in American couples’ smoking behaviour when facing health shocks, and found that men are more likely to benefit from their partners rather than the other way around. We therefore test if any differences between men and women exist regarding the association between living arrangements and illicit drug use.

### Research Questions and Research Hypotheses

Taking living arrangements as a type of collectivity through the lens of the health lifestyle theory, our research aims to address two research questions. First, whether or not young, middle-aged, and older Canadians’ living arrangements (living alone vs. living with spouses/partners, children, both, or others) are associated with their illicit drug use, and if so, to what magnitude? The reason for dividing respondents into three age groups lies in the fact that living with parents, spouses/partners, children, or others may have different social, emotional, and health meanings depending on one’s age and different life stages (31, 32). To answer the first question, we propose Hypothesis 1 (H1) considering the complex associations between living arrangements and people’s health behaviours.

H1 Canadians living alone are more likely to use illicit drugs compared to those co-residing with core family members.

Second, are there any differences between men and women in the associations between living arrangements and illicit drug use? Prior studies indicated that men are more likely to receive positive influences from their female partners regarding changes in health behaviours (28, 30). Hypothesis 2 (H2) has thus been proposed in the current study to address whether sex difference exists in relevance to our core research interest.

H2 The difference between living alone and living with families regarding illicit drug use is more significant among women than men.

## Methods

### Data

We use the 2015–2016 Canadian Community Health Survey (CCHS) Annual Component. Statistics Canada collected, administrated, and released the data. The CCHS is a series of repeated cross-sectional datasets focusing on the physical and mental health conditions of Canadian residents aged 12 and above. The 2015–2016 CCHS collected data on Canadians’ illicit drug use, living arrangements, demographic and socioeconomic backgrounds, and physical and mental health statuses, with an acceptable proportion of missing values in total. Such data richness ensures successful modelling to address research questions. The CCHS 2015 is also nationally representative (33), guaranteeing the generalizability of research findings.

### Sample

This article focuses on Canadians aged 20 and above. The reason is that, according to CCHS 2015, Canadians aged 12–19 rarely lived by themselves (0.65%). We further divided Canadians aged 20 and above into three age groups: “young Canadians” or “young adults” who were aged 20–34 years, “middle-aged Canadians” or “middle-aged adults” aged 35–59 years, and “older Canadians” or “older adults” aged 60 and above. We deleted samples with missing values which are 3.22%, 3.84%, and 5.89% of the total sample size of the three age groups, respectively. The sample size of young Canadians is 19,091, and the sample sizes of middle-aged Canadians and older Canadians are 38,241 and 36,863, respectively. The final total sample size is 94,195.

### Measures

#### The Dependent Variable

The variable of recent use of illicit drugs focuses on whether the respondent used illicit drugs in the past 12 months. It is coded dichotomously, namely “yes” and “no.”

#### The Key Predictor: Living Arrangements

It is notable that living arrangements contain various categories. Quantitative studies usually code living arrangements into a dummy or multiple-categorical variable to examine whether one specific living arrangement differs from others in predicting health outcomes. If living arrangements have been dichotomously coded, the variable usually includes “living alone” and “living with others” (17, 34). “Living with others” can be further divided into multiple categories based on whom the respondent lives with, including partners/spouses, children, siblings, relatives, others, and so forth (19, 35). Also, scholars measure whether a respondent lives apart together by tabulating married status and living arrangements (36).

CCHS 2015 asked respondents about their “living/family arrangement of selected respondent.” For young adults, we coded their living arrangements into six categories: “living alone,” “living with spouses/partners,” “living with spouses/partners and children,” “living with children only,” “living with parents,” and “other types.” The last category includes those “unattached respondents living with others” and “other”. As for middle-aged and older Canadians, their living arrangements were coded into five categories without “living with parents” due to small proportions of this type. We thus combined “living with parents” with “other types.”

#### Covariates

Three sets of covariates were added as control variables, including respondents’ demographic background, socioeconomic status, and health conditions. Demographic background includes age groups, sex, racial background, immigrant status, and province of residence. Socioeconomic status has three indicators: educational attainment, personal income levels, and dwelling ownership. Health conditions include whether a respondent has any type of chronic disease. Detailed measurements of the covariates are upon request.

### Weighted Sample Characteristics

Table 1 presents weighted sample characteristics with a 95% Confidence Interval (CI). The proportion of respondents living alone is 26.83% of older Canadians, the highest among all three, followed by middle-aged Canadians’ 13.17% and young Canadians’ 11.23%. Similarly, older Canadians have the highest proportion of living with spouses/partners (53.52%), which is about 32% higher than the proportion of middle-aged Canadians (21.04%) and 36% higher than that of young Canadians (17.47%) with statistical significance. Middle-aged Canadians have the largest proportion of living with spouses/partners and children (46.47%), which is more than twice higher compared to young Canadians (22.10%) and approximately six times higher than that of older adult Canadians (8.10%). These differences are statistically significant as indicated by their nonoverlapping 95% CIs. As for the proportion of living with children only, the middle-aged group has a significantly higher proportion (7.18%) than their young (2.89%) and older counterparts (2.93%). Lastly, the proportion of living in other types of arrangements is the highest among young adults (19.26%), which is about 7% higher in comparison with the middle-aged (12.14%) and about 10% higher than older adults (8.62%) with statistical significance. Thus, noticeably, that young, middle-aged, and older Canadians significantly differ in the distributions of living arrangement types, which demonstrates the reasonability of separate modelling for young, middle-aged, and older Canadians.

**TABLE 1:** Weighed sample characteristics (%) with 95% Confidence Interval of young (20–34), middle-aged (35–59), and older Canadians (60+), Canadian Community Health Survey (Canada. 2015–2016).

Independent variables	Young (N = 19,091)	Middle-aged (N = 38,241)	Older (N = 36,863)
Living arrangements			
Living alone	11.23 (10.69, 11.79)	13.17 (12.76, 13.59)	26.83 (26.20, 27.47)
With spouses/partners	17.47 (16.62, 18.36)	21.04 (20.41, 21.67)	53.52 (52.64, 54.40)
With spouses/partners and children	22.10 (21.23, 22.99)	46.47 (45.59, 47.36)	8.10 (7.45, 8.81)
With children only	2.89 (2.62, 3.18)	7.18 (6.74, 7.66)	2.93 (2.60, 3.31)
With parents	27.05 (25.93, 28.21)	n.a.	n.a.
Other types	19.26 (18.22, 20.33)	12.14 (11.48, 12.83)	8.62 (7.96, 9.31)
Age groups			
20–24	30.27 (29.15, 31.42)		
25–29	32.00 (30.91, 33.11)		
30–34	37.72 (36.61, 38.85)		
35–39		18.34 (17.70, 19.01)	
40–44		18.95 (18.28, 19.64)	
45–49		20.54 (19.78, 21.31)	
50–54		21.09 (20.38, 21.82)	
55–59		21.08 (20.40, 21.77)	
60–64			30.39 (29.52, 31.27)
65–69			26.67 (25.91, 27.45)
70–74			18.45 (17.82, 19.10)
75–79			12.05 (11.56, 12.56)
80+			12.43 (11.96, 12.93)
Sex			
Male	49.90 (48.72, 51.08)	49.63 (48.75, 50.51)	46.22 (45.35, 47.09)
Female	50.10 (48.92, 51.28)	50.37 (49.49, 51.25)	53.78 (52.91, 54.65)
Racial background			
White	64.76 (63.53, 65.97)	72.27 (71.38, 73.14)	82.84 (82.00, 83.65)
Racial minorities	32.04 (30.84, 33.26)	23.84 (23.01, 24.70)	12.95 (12.20, 13.74)
Missing	3.20 (2.78, 3.68)	3.89 (3.49, 4.33)	4.21 (3.82, 4.63)
Immigrant status			
Canadian born	73.14 (71.94, 74.30)	69.60 (68.68, 70.49)	71.82 (70.92, 72.71)
Landed immigrant	23.57 (22.43, 24.74)	26.43 (25.55, 27.32)	24.10 (23.24, 24.98)
Missing	3.30 (2.88, 3.78)	3.98 (3.59, 4.41)	4.08 (3.70, 4.49)
Province of residence			
Ontario	38.54 (37.32, 39.77)	38.27 (37.36, 39.18)	37.80 (36.89, 38.72)
Quebec	22.06 (21.13, 23.03)	23.56 (22.84, 24.29)	25.75 (25.03, 26.48)
BC	12.70 (11.98, 13.45)	12.93 (12.40, 13.48)	13.59 (13.05, 14.14)
Alberta	13.77 (13.09, 14.49)	12.09 (11.59, 12.62)	8.79 (8.36, 9.24)
Other provinces and territories	12.93 (12.34, 13.54)	13.15 (12.73, 13.59)	14.08 (13.64, 14.52)
Educational attainment			
Elementary school and below	6.43 (5.95, 6.95)	8.37 (7.91, 8.85)	21.58 (20.92, 22.26)
Secondary and high school	24.65 (23.65, 25.69)	20.24 (19.58, 20.91)	22.68 (21.95, 23.43)
College and above	68.92 (67.83, 69.99)	71.39 (70.62, 72.15)	53.64 (52.78, 54.51)
Missing	n.a.	n.a.	2.09 (1.84, 2.38)
Personal income			
<20,000	34.76 (33.61, 35.93)	17.93 (17.28, 18.60)	25.61 (24.86, 26.38)
20,000–39,999	25.04 (24.08, 26.03)	20.10 (19.43, 20.79)	29.27 (28.50, 30.06)
40,000–59,999	15.91 (15.12, 16.74)	19.41 (18.73, 20.10)	16.89 (16.25, 17.55)
60,000–79,999	9.31 (8.69, 9.97)	13.29 (12.72, 13.87)	8.37 (7.92, 8.86)
80,000+	8.76 (8.17, 9.39)	22.65 (21.92, 23.40)	10.66 (10.11, 11.24)
Missing	6.21 (5.55, 6.94)	6.63 (6.10, 7.19)	9.20 (8.67, 9.75)
Dwelling ownership			
Owned	58.88 (57.71, 60.05)	74.20 (73.39, 74.98)	75.42 (74.64, 76.18)
Rent	38.28 (37.13, 39.44)	22.16 (21.43, 22.91)	20.57 (19.88, 21.29)
Missing	2.84 (2.43, 3.31)	3.64 (3.25, 4.08)	4.01 (3.62, 4.44)
Has chronic disease or not			
No chronic disease	84.57 (83.73, 85.37)	63.23 (62.39, 64.06)	27.70 (26.91, 28.49)
Has at least one chronic disease	15.43 (14.63, 16.27)	36.77 (35.94, 37.61)	72.30 (71.51, 73.09)

Note. “n.a.” refers to “not available."

### Statistical Analysis

We applied two regression models. Binary logit regression was applied to predict whether using illicit drugs in the past 12 months for young and middle-aged adults because the dependent variable is dichotomously coded. But for predicting illicit drug use during the past year among older adults, the complementary log-log model was used because the proportion of illicit drug use is relatively small among the aged population (37). Our multivariable modelling logic includes two steps. In the first step, we began by adding the independent variable and all covariates to the model to identify the association between living arrangements and illicit drug use among young, middle-aged, and older Canadians. In the second step, we further added the interaction term between living arrangements and sex to the model to test possible sex differences in this vein.

## Results

**FIGURE 1:**
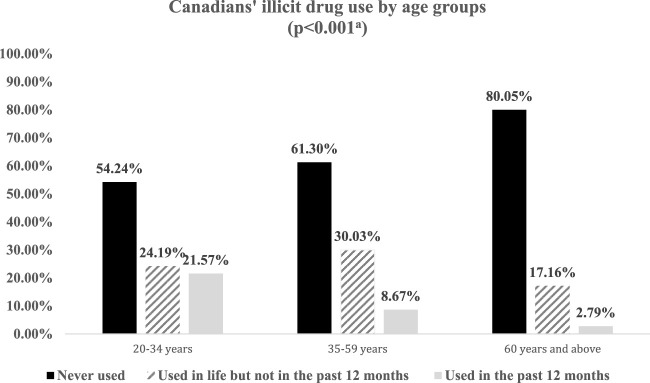
Weighted proportions of Canadians’ illicit drug use by age groups (N = 94,195), Canadian Community Health Survey (Canada. 2015–2016). *Note.* a: The result of the corrected weighted Pearson Chi^2^ shows that Canadians’ illicit drug use and age groups are significantly correlated (*p* < 0.001). This is in consistent with bivariate results based on binary logit regression using illicit drug use as the dependent variable that, compared to young adults aged 20–34, middle-aged Canadians (OR 0.34, 95% CI: 0.32–0.37, *p* < 0.001) and older Canadians (OR 0.10, 95% CI: 0.09–0.12, *p* < 0.001) are substantially less likely to use illicit drugs.

Figure 1 presents Canadians’ illicit drug use by age groups. As shown, older Canadians have the highest proportion (80.05%) of never using illegal drugs over their life course. In comparison, the proportions of middle-aged and young Canadians who have never done so are 61.30% and 54.24%, respectively. Middle-aged Canadians have the highest proportion (30.03%) regarding having used illicit drugs, but not in the past 12 months, compared to their younger (24.19%) and older (17.16%) counterparts. When it comes to the proportion of those using illicit drug use in the last year, young Canadians have a significantly higher proportion (21.57%) than middle-aged (8.67%) and older (2.79%) Canadians. The weighted Pearson statistic indicates a substantial difference in illicit drug use due to age groups (*p* < 0.001). This association between age and using illegal drugs also confirms the cohort difference that illicit drug use is more prevalent among younger cohorts than older ones.

Table 2 presents bivariate associations between living arrangements and illicit drug use. The weighted corrected Pearson Chi^2^ test results show that the two variables are significantly correlated (*p* < 0.001) for all three age groups. For young Canadians, about 28.55% of those solo-living respondents used illicit drugs in the past 12 months. This proportion is about 18% higher than their counterparts living with spouses/partners and children, 10% higher than those living with children only, and 7% higher than those living with spouses/partners. But the proportion is not significantly different from their peers living with parents and living in other types of arrangements, as indicated by overlapped 95% CIs. Similarly, the proportion of middle-aged Canadians who used illicit drugs is the highest among the solo-living ones (15.91%), which is approximately three times higher than those living with spouses/partners with children and about twice that of those living with spouses/partners only. It is also about 5% higher compared to those living with children only and 4% higher compared to those living in other types of arrangements with statistical significance. As for older Canadians, the proportion using illicit drugs, despite being small, is still higher among those living alone (3.39%) compared to those living with spouses/partners only (2.34%) and those living with children only (0.99%). These differences are statistically significant, according to the nonoverlapping 95% CIs. But older adults living alone do not differ from their peers who live with spouses/partners and children concerning the proportion of illicit drug use. In brief, for all three age groups, Canadian adults living alone have reported higher proportions of illicit drug use during the past 12 months than many of their counterparts living with core family members.

**TABLE 2:** Weighted proportions with 95% Confidence Interval of illicit drug use among Canadians young, middle-aged, and older adults by their living arrangements, Canadian Community Health Survey (Canada. 2015–2016).

	Illicit drug use (%)		
	Never used	Once used but not in the past 12 months	Used in the past 12 months
Living arrangements among young Canadians aged 20–34 (N = 19,091)			
Living alone	46.51 (44.09, 48.96)	24.94 (22.86, 27.14)	28.55 (26.40, 30.80)
With spouses/partners	47.88 (45.17, 50.61)	30.53 (28.15, 33.02)	21.59 (19.21, 24.17)
With spouses/partners and children	55.37 (53.25, 57.48)	33.80 (31.86, 35.80)	10.82 (9.52, 12.27)
With children only	48.44 (43.61, 53.30)	33.33 (29.19, 37.75)	18.23 (14.98, 22.00)
With parents	61.01 (58.52, 63.44)	13.46 (11.94, 15.14)	25.53 (23.38, 27.80)
Other types	54.56 (51.46, 57.61)	20.67 (18.38, 23.17)	24.77 (22.38, 27.32)
Living arrangements among middle-aged Canadians aged 35–59 (N = 38,241)			
Living alone	49.93 (48.39, 51.46)	34.17 (32.73, 35.63)	15.91 (14.89, 16.99)
With spouses/partners	57.52 (55.91, 59.11)	33.69 (32.16, 35.26)	8.79 (7.89, 9.77)
With spouses/partners and children	66.53 (65.24, 67.78)	28.12 (26.93, 29.35)	5.35 (4.84, 5.92)
With children only	57.13 (53.87, 60.33)	31.76 (28.87, 34.80)	11.10 (9.32, 13.19)
Other types	62.66 (59.75, 65.47)	25.49 (23.04, 28.12)	11.85 (10.14, 13.81)
Living arrangements among older Canadians aged 60+ (N = 36,863)			
Living alone	79.91 (78.96, 80.83)	16.70 (15.83, 17.59)	3.39 (3.01, 3.82)
With spouses/partners	80.81 (79.94, 81.66)	16.84 (16.04, 17.67)	2.34 (2.04, 2.69)
With spouses/partners and children	75.15 (71.16, 78.75)	22.18 (18.69, 26.12)	2.67 (1.80, 3.94)
With children only	84.71 (79.81, 88.60)	14.30 (10.49, 19.20)	0.99 (0.48, 2.02)
Other types	78.73 (74.84, 82.17)	16.87 (13.74, 20.54)	4.40 (2.89, 6.63)

In Table 3, we ran multivariable regressions to examine associations between living arrangements and illicit drug use of Canadian adults. Models 1a, 2a, and 3a control for all covariates, and Models 1b, 2b, and 3b further test differences by adding the interaction term between sex and living arrangements to the model. Related test results show that VIF values range between 1.19 and 1.24, indicating that no multicollinearity issues exist in these multivariable regressions.

**TABLE 3:** Odds ratios with 95% Confidence Interval from binary logit regressions and coefficients complimentary log-log regressions predicting illicit drug use in the past 12 months among Canadians aged 20+, Canadian Community Health Survey (Canada. 2015–2016).

	Young (N = 19,091)		Middle-aged (N = 38,241)		Older (N = 36,863)	
	Model 1a	Model 1b	Model 2a	Model 2b	Model 3a	Model 3b
Living arrangements (Living alone)
With spouses/partners	0.77**(0.64, 0.94)	0.81 (0.63, 1.06)	0.69*** (0.59, 0.82)	0.76** (0.62, 0.93)	−0.55*** (−0.76, −0.34)	−0.57*** (−0.81, −0.33)
With spouses/partners and children	0.36*** (0.29, 0.43)	0.50*** (0.39, .64)	0.38*** (0.33, 0.44)	0.44*** (0.37, 0.53)	−0.66** (−1.10, −0.22)	−0.63* (−1.11, −0.15)
With children only	0.57*** (0.44, 0.75)	0.83 (0.46, 1.51)	0.92 (0.74, 1.15)	1.20 (0.83, 1.72)	−1.07** (−1.83, −0.30)	−0.89* (−1.75, −0.03)
With parents	0.96 (0.79, 1.18)	0.97 (0.77, 1.22)	n.a	n.a.	n.a.	n.a.
Other types	0.95 (0.79, 1.15)	1.05 (0.83, 1.32)	0.80* (0.66, 0.98)	0.93 (0.72, 1.19)	0.07 (−0.43, 0.56)	−0.004 (−0.60, 0.59)
Sex (Male)						
Female	0.53*** (0.46, 0.60)	0.66*** (0.52, 0.83)	0.42*** (0.37, 0.47)	0.57*** (0.48, 0.68)	−1.23*** (−1.45, −1.00)	−1.27*** (−1.52, −1.01)
Living arrangements # Sex (Living alone # Female)						
With spouses/partners # Female		0.86 (0.59, 1.26)		0.78 (0.57, 1.04)		0.06 (−0.35, 0.48)
With spouses/partners and children # Female		0.42*** (0.29, 0.61)		0.63** (0.47, 0.85)		−0.28 (−1.32, 0.76)
With children only # Female		0.56 (0.29, 1.10)		0.57* (0.36, 0.90)		−0.55 (−2.50, 1.41)
With parents # Female		0.95 (0.68, 1.33)		n.a.		n.a.
Other types # Female		0.76 (0.53, 1.09)		0.64* (0.43, 0.95)		0.22 (−0.73, 1.18)
Pseudo *R* ^2^	0.0870	0.0896	0.1111	0.1122	n.a.	n.a.
Log pseudo-likelihood	−3311868.5	−3302655.8	−3091770.2	−3088160.4	−801360.29	−801074.53

*Note*. *p < 0.05; **p < 0.01; ***p < 0.001. “#” is the symbol of interaction terms. “n.a.” refers to “not available.” All models control for respondents’ age group, racial background, immigrant status, province of residence, educational attainment, personal income, dwelling ownership, and chronic disease status. Results are available based on requests.

Model 1a shows that, compared to their counterparts living alone, young adults living with spouses/partners are about 20% less likely to use illicit drugs in the past 12 months (*p* < 0.01). Those living with spouses/partners and children are more than 60% less likely to use illicit drugs (*p* < 0.001), and those living with children only are about 40% less likely to do so (*p* < 0.001). However, for young Canadians, living with parents does not differ in using illicit drugs compared to living alone. Results of Model 1b further indicate that statistically significant differences exist in the relationship between young Canadians’ living arrangements and drug use due to sex. In particular, living with spouses/partners and children is more favourable to young women than men, as their likelihood of using illicit drugs is more than half lower compared to their solo-living counterparts (*p* < 0.001).

Models 2a and 2b present situations for middle-aged Canadians. Model 2a shows that spouses/partners and children both play leading roles in middle-aged Canadians’ households. Specifically, living with spouses/partners with children or without children are both associated with significantly lower odds of using illicit drugs (*p* < 0.001). Model 2b indicates that significant differences owing to sex exist in women who live with children, either with spouses/partners (*p* < 0.01) or not (*p* < 0.05). They are both about 40% less likely to use illicit drugs than those living independently.

As for older Canadians, as Model 3a presents, older adults living with spouses/partners and/or children were substantially less likely to have used illicit drugs than their solo-living peers (*p* < 0.01 or *p* < 0.001). The coefficient disparities show that adult children play the most significant role in preventing older Canadians from using illicit drugs. However, the likelihood of illicit drug use did not differ between older adults living alone or in settings with other non-family individuals. Further, Model 3b demonstrates no difference between older Canadian men and women in the association between living arrangements and using illicit drugs.

## Discussion

This article aims to identify the possible relationship between Canadians’ living arrangements and illicit drug use, an unhealthy and deviant behaviour among Canadian adults (2). Findings first indicate a close relationship between Canadians’ living arrangements (living alone versus other types of living arrangements) and their use of illicit drugs, assuming respondents’ living arrangements did not change in the past year before the survey time. The association can be complex through the theoretical lens of the health lifestyle theory, which highlights the importance of collectivity that may affect an individual’s health behaviours and health lifestyles (22, 23).

Two findings related to the main research question should be emphasized. The first finding supports Hypothesis 1 that living alone should be considered a risk factor for all three age groups. Living with core family members generally contributes to higher likelihoods of not using illicit drugs. Second, we argue that living arrangements can be seen as a type of collectivity through the perspective of the health lifestyle theory (38). Findings show that living with different types of family members function differently in preventing people from using illicit drugs. For young Canadians, their children play the most positive role within the household, followed by their spouses/partners. This is similar to older Canadians, whose adult children may have exerted the most significant positive influences on lowering their likelihoods of using illicit drugs. Spouses/partners also play a crucial part in older Canadians’ households on this matter. As for middle-aged Canadians, spouses/partners play the most positive roles in their health behaviours and lifestyles. Worth noting is that their co-residing children did not take an active part in this regard.

It is notable that we did not assume that illicit drug use only occurs in households. Although respondents may have used illicit drugs outside rather than within their households, co-residing with families can still exert a strong influence on the development of people’s health behaviours through daily interactions, especially compared to those living alone. In the current research, data limitations make it impossible to address the social contexts of respondents’ initiation and consistent use of illicit drugs (e.g., under peer pressure). Despite this shortcoming, living arrangements still take an active part in Canadians’ illicit drug use from the theoretical perspective of health lifestyle theory.

Findings also address another critical research question: Do differences between men and women exist regarding the association between Canadians’ living arrangements and illicit drug use? We found that spouses/partners and children work as stronger bonds for young and middle-aged Canadian women than for men, supporting Hypothesis 2. This may be because women are less willing to take risks using illicit drugs, especially when living with their children, indicating a significant sex difference.

### Implications

The policy implications are apparent in the context that an increasing number of Canadians now live by themselves. We first recommend that the local communities and governments emphasize solo-living Canadians who are likely to be put into a risky situation regarding using illicit drugs, especially for young and middle-aged men.

Additionally, cohort differences should also be taken into consideration because the younger cohorts in this study, or the “Generation Y” (also known as Millennials), who were born in the 1980s and the first half of the 1990s, have significantly higher proportions and likelihoods of using illicit drugs in the past 12 months in comparison to the middle-aged and older cohorts. Importantly, if these young adults lack significant co-residing members (spouses/partners and children) who may play a positive role as a “supervisor” on their health behaviours and health lifestyles within the household, they have an even higher risk of engaging in these behaviours.

### Limitations and Future Directions

This study has a couple of limitations to report. First, the main limitation is that the dependent variable—whether a respondent used illicit drugs during the past year—is broad. It remains unclear what types of illicit drugs respondents have used and their frequency.

Second, endogeneity issues remain. Causality has not been identified due to relevant data limitations. However, two-way causality may exist. Specifically, using illicit drugs may have contributed to higher risks of marital disruption, which have led to the shifts in respondents’ living arrangements. Tracking people’s living arrangement transitions may help in addressing this issue.

Third, respondents’ interactions with families (within households) and peers (outside households) are unknown. However, stronger interactions within households may increase the likelihood of respondents’ recent illicit drug use if their family members were drug users. Similarly, having frequent interactions with peers who perform deviant behaviours significantly predict young adults’ drug use behaviours (25). Therefore, respondents’ interactions with families and peers deserve more exploration when investigating their illicit drug use possibilities.

The last limitation is that unattached respondents living with others were coded into the category of “other types.” Those unattached respondents have thus not been analysed in detail. Closer attention should address who they are and the underlying reasons for their illicit drug use, since this subpopulation reported high proportions of doing so, especially among the young and middle-aged.

### Conclusion

Our research is the pioneer in investigating the multifaceted associations between living arrangements and Canadians’ recent illicit drug use behaviour. According to our findings, the first crucial take-home message demonstrates that living alone can be a risk factor for Canadians’ use of illicit drugs. Second, importantly, children and spouses/partners may have positively prevented young and older Canadians from using illicit drugs within the household. Living with spouses/partners works the most positively for middle-aged Canadians. Lastly, differences owing to sex have also been found regarding illicit drug use, in that living with families may have a more positive effect on young and middle-aged women compared to men. Policy recommendations are proposed accordingly that more health-related support should be provided to Canadians living by themselves.

## References

[1] Government of Canada. Cannabis Legalization and Regulation (2021). Available at: https://www.justice.gc.ca/eng/cj-jp/cannabis/ (Accessed July 07, 2021).

[2] Statistics Canada. Alcohol and Drug Use in Canada, 2019 (2021). Available at: https://www150.statcan.gc.ca/n1/daily-quotidien/211220/dq211220c-eng.pdf (Accessed December 20, 2021).

[3] Elflein J. Drug Use in Canada - Statistics & Facts. Statista (2022). Available at: https://www.statista.com/topics/4533/drug-use-in-canada/#topicHeader__wrapper (Accessed January 20, 2022).

[4] Macleod J Oakes R Copello A Crome I Egger M Hickman M Psychological and Social Sequelae of Cannabis and Other Illicit Drug Use by Young People: A Systematic Review of Longitudinal, General Population Studies. Lancet (2004) 363(9421):1579–88. 10.1016/S0140-6736(04)16200-4 15145631

[5] Fischer B Murphy Y Rudzinski K MacPherson D. Illicit Drug Use and Harms, and Related Interventions and Policy in Canada: a Narrative Review of Select Key Indicators and Developments since 2000. Int J Drug Pol (2016) 27:23–35. 10.1016/j.drugpo.2015.08.007 26359046

[6] Cofrancesco J Jr Scherzer R Tien PC Gibert CL Southwell H Sidney S Illicit Drug Use and HIV Treatment Outcomes in a US Cohort. AIDS (London, England) (2008) 22(3):357–65. 10.1097/QAD.0b013e3282f3cc21 18195562 PMC3189479

[7] Beynon CM Roe B Duffy P Pickering L. Self Reported Health Status, and Health Service Contact, of Illicit Drug Users Aged 50 and over: a Qualitative Interview Study in Merseyside, United Kingdom. BMC Geriatr (2009) 9(1):45–9. 10.1186/1471-2318-9-45 19818114 PMC2763853

[8] Rehm J Room R van den Brinkt W Kraus L. Problematic Drug Use and Drug Use Disorders in EU Countries and Norway: An Overview of the Epidemiology. Eur Neuropsychopharmacol (2005) 15(4):389–97. 10.1016/j.euroneuro.2005.04.004 15955677

[9] Liu L Chai X. Pleasure and Risk: A Qualitative Study of Sexual Behaviors Among Chinese Methamphetamine Users. J Sex Res (2020) 57(1):119–28. 10.1080/00224499.2018.1493083 30004801

[10] Young M Stuber J Ahern J Galea S. Interpersonal Discrimination and the Health of Illicit Drug Users. The Am J Drug Alcohol Abuse (2005) 31(3):371–91. 10.1081/ada-200056772 16161724

[11] Heydari ST Izedi S Sarikhani Y Kalani N Akbary A Miri A The Prevalence of Substance Use and Associated Risk Factors Among university Students in the City of Jahrom, Southern Iran. Int J High Risk Behaviors Addict (2015) 4(2):e22381. 10.5812/ijhrba.4(2)2015.22381 PMC4464575 26097836

[12] Ilhan IÖ Yıldırım F Demirbaş H Doğan YB. Prevalence and Sociodemographic Correlates of Substance Use in a university-student Sample in Turkey. Int J Public Health (2009) 54(1):40–4. 10.1007/s00038-009-7049-1 19142577

[13] El Ansari W Vallentin-Holbech L Stock C. Predictors of Illicit Drug/s Use Among university Students in Northern Ireland, Wales and England. Glob J Health Sci (2015) 7(4):18–29. 10.5539/gjhs.v7n4p18 PMC4802112 25946914

[14] Kim N Kim H Kwon S. Factors Associated with Different Numbers of Health Behaviors by Living Arrangements. BMC Public Health (2020) 20(1):1141–11. 10.1186/s12889-020-09242-y 32689961 PMC7372790

[15] Hanna KL Collins PF. Relationship between Living Alone and Food and Nutrient Intake. Nutr Rev (2015) 73(9):594–611. 10.1093/nutrit/nuv024 26269488

[16] Zhang J Wu L. Cigarette Smoking and Alcohol Consumption Among Chinese Older Adults: Do Living Arrangements Matter? Int J Environ Res Public Health (2015) 12(3):2411–36. 10.3390/ijerph120302411 25711361 PMC4377909

[17] Chai X. Living Alone: Five Decades of Change, and its Implications for Health. Electronic Thesis and Dissertation Repository. London: University of Western Ontario (2019): 6462.

[18] Helliwell JF Schellenberg G Fonberg J. Life Satisfaction in Canada before and during the COVID-19 Pandemic. In: Analytical Studies Branch Research Paper Series. Statistics Canada (2020). Available at: https://www150.statcan.gc.ca/n1/pub/11f0019m/11f0019m2020020-eng.pdf .

[19] Chai X Margolis R. Does Living Alone Mean Spending Time Differently? Time Use and Living Arrangements Among Older Canadians. Can Stud Popul (2020) 47(1):9–25. 10.1007/s42650-020-00017-9

[20] Statistics Canada. Families, Households and Marital Status: Key Results from the 2016 Census (2017). Available at: https://www150.statcan.gc.ca/n1/daily-quotidien/170802/dq170802a-eng.htm (Accessed August 02, 2017).

[21] Tang J Galbraith N Truong J. Living Alone in Canada (2019). Available at: https://www150.statcan.gc.ca/n1/pub/75-006-x/2019001/article/00003-eng.pdf (Accessed March 06, 2019).

[22] Cockerham WC. Health Lifestyle Theory in an Asian Context. Health Sociol Rev (2006) 15(1):5–15. 10.5172/hesr.2006.15.1.5

[23] Cockerham WC. Health Lifestyle Theory and the Convergence of agency and Structure. J Health Soc Behav (2005) 46(1):51–67. 10.1177/002214650504600105 15869120

[24] Cockerham WC Rütten A Abel T. Conceptualizing Contemporary Health Lifestyles: Moving beyond Weber. Sociological Q (1997) 38(2):321–42. 10.1111/j.1533-8525.1997.tb00480.x

[25] Norman LB Ford JA. Adolescent Ecstasy Use: A Test of Social Bonds and Social Learning Theory. Deviant Behav (2015) 36(7):527–38. 10.1080/01639625.2014.944072

[26] Tucker JS Anders SL. Social Control of Health Behaviors in Marriage 1. J Appl Soc Psychol (2001) 31(3):467–85. 10.1111/j.1559-1816.2001.tb02051.x

[27] Tani Y Kondo N Takagi D Saito M Hikichi H Ojima T Combined Effects of Eating Alone and Living Alone on Unhealthy Dietary Behaviors, Obesity and Underweight in Older Japanese Adults: Results of the JAGES. Appetite (2015) 95:1–8. 10.1016/j.appet.2015.06.005 26116391

[28] Duncan GJ Wilkerson B England P. Cleaning up Their Act: The Effects of Marriage and Cohabitation on Licit and Illicit Drug Use. Demography (2006) 43(4):691–710. 10.1353/dem.2006.0032 17236542

[29] Helgeson VS Novak SA Lepore SJ Eton DT. Spouse Social Control Efforts: Relations to Health Behavior and Well-Being Among Men with Prostate Cancer. J Soc Personal Relationships (2004) 21(1):53–68. 10.1177/0265407504039840

[30] Margolis R. Health Shocks in the Family: Gender Differences in Smoking Changes. J Aging Health (2013) 25(5):882–903. 10.1177/0898264313494411 23860178 PMC3863597

[31] Bures RM. Living Arrangements over the Life Course: Families in the 21st century. J Fam Issues (2009) 30(5):579–85. 10.1177/0192513x08331131

[32] Zueras P Rutigliano R Trias-Llimós S. Marital Status, Living Arrangements, and Mortality in Middle and Older Age in Europe. Int J Public Health (2020) 65(5):627–36. 10.1007/s00038-020-01371-w 32350551 PMC7360666

[33] Statistics Canada. Canadian Community Health Survey - Annual Component (CCHS) (2016). Available at: https://www23.statcan.gc.ca/imdb/p2SV.pl?Function=getSurvey&Id=238854 (Accessed June 24, 2016).

[34] Chou KL Chi I. Comparison between Elderly Chinese Living Alone and Those Living with Others. J Gerontological Soc Work (2000) 33(4):51–66. 10.1300/j083v33n04_05

[35] Alwin DF Converse PE Martin SS. Living Arrangements and Social Integration. J Marriage Fam (1985) 47:319–34. 10.2307/352132

[36] Hughes ME Waite LJ. Health in Household Context: Living Arrangements and Health in Late Middle Age. J Health Soc Behav (2002) 43(1):1–21. 10.2307/3090242 11949193 PMC1440422

[37] Penman AD Johnson WD. Complementary Log–Log Regression for the Estimation of Covariate‐adjusted Prevalence Ratios in the Analysis of Data from Cross‐sectional Studies. Biom J J Math Methods Biosci (2009) 51(3):433–42. 10.1002/bimj.200800236 19588454

[38] Chai X Mei J. Investigating Food Insecurity, Health Lifestyles, and Self-Rated Health of Older Canadians Living Alone. BMC Public Health (2022) 22(1):2264. 10.1186/s12889-022-14467-0 36464679 PMC9720941

